# Challenges and opportunities in evaluating programmes incorporating human-centred design: lessons learnt from the evaluation of Adolescents 360

**DOI:** 10.12688/gatesopenres.12998.2

**Published:** 2019-09-25

**Authors:** Aoife M. Doyle, Emma Mulhern, James Rosen, Gabrielle Appleford, Christina Atchison, Christian Bottomley, James R. Hargreaves, Michelle Weinberger

**Affiliations:** 1 London School of Hygiene & Tropical Medicine, London, WC1E7HT, UK; 2 Itad Ltd, Hove, BN31RE, UK; 3 Avenir Health, Washington, DC 20012, USA

**Keywords:** Human-Centered Design, User-centered Design, Design Thinking, Evaluation, Adolescents, Contraception, Africa

## Abstract

Adolescents 360 (A360) is a four-year initiative (2016–2020) to increase 15-19-year-old girls’ use of modern contraception in Nigeria, Ethiopia and Tanzania. The innovative A360 approach is led by human-centred design (HCD), combined with social marketing, developmental neuroscience, public health, sociocultural anthropology and youth engagement ‘lenses’, and aims to create context-specific, youth-driven solutions that respond to the needs of adolescent girls. The A360 external evaluation includes a process evaluation, quasi-experimental outcome evaluation, and a cost-effectiveness study. We reflect on evaluation opportunities and challenges associated with measuring the application and impact of this novel HCD-led design approach.

For the process evaluation, participant observations were key to capturing the depth of the fast-paced, highly-iterative HCD process, and to understand decision-making within the design process. The evaluation team had to be flexible and align closely with the work plan of the implementers. The HCD process meant that key information such as intervention components, settings, and eligible populations were unclear and changed over outcome evaluation and cost-effectiveness protocol development. This resulted in a more time-consuming and resource-intensive study design process. As much time and resources went into the creation of a new design approach, separating one-off “creation” costs versus those costs associated with actually implementing the programme was challenging. Opportunities included the potential to inform programmatic decision-making in real-time to ensure that interventions adequately met the contextualized needs in targeted areas.

Robust evaluation of interventions designed using HCD, a promising and increasingly popular approach, is warranted yet challenging. Future HCD-based initiatives should consider a phased evaluation, focusing initially on programme theory refinement and process evaluation, and then, when the intervention program details are clearer, following with outcome evaluation and cost-effectiveness analysis. A phased approach would delay the availability of evaluation findings but would allow for a more appropriate and tailored evaluation design.

## Rationale

In the field of public health, human-centred design (HCD), also known as design thinking, is increasingly being used during intervention development, and sometimes implementation, to design innovative solutions to complex problems ^1^. HCD is a creative approach to programme design that focuses on building empathy with the population of interest, and generating and iteratively testing many potential solutions to complex problems. The approach has a high tolerance for both ambiguity and failure ^1– 3^. HCD shares some characteristics with other methods used to design programmes such as traditional socio-behavioural research but tends to be more ‘nimble/flexible/iterative’ and less protocol driven ^4^.

Intervention evaluation is an essential component of public health research and programming, and detailed guidance on evaluation approaches and methodologies exist ^5, 6^. However, the iterative and flexible nature of HCD potentially poses some unique challenges for evaluation ^1, 3, 7^ and examples of evaluations of HCD public health interventions have so far been limited ^7, 8^. There are some parallels between evaluations of HCD interventions and evaluations of interventions incorporating adaptive management or quality improvement where the intervention can also change over time ^9– 14^. Also, programmatic interventions, as opposed to researcher-led interventions, often involve an element of flexibility in intervention implementation, as implementers strive to develop more context appropriate interventions, and respond to programmatic targets. In such interventions, the location and timing of intervention implementation is guided primarily by programme targets or local government priorities, and less so by a need to maximise the quality of evidence of impact ^15, 16^. Despite some overlap with other evaluation scenarios, we have identified some unique challenges to the evaluation of HCD-led interventions.

## Purpose of the letter

In this letter we discuss the evaluation opportunities and challenges identified during the ongoing A360 evaluation and make recommendations for future evaluations of programmes designed using HCD.

## Adolescents 360

The intervention that is under evaluation is Adolescents 360 (A360), a multi-country initiative which aims to increase the voluntary use of modern contraceptives among adolescent girls aged 15–19 years. The A360 initiative is led by Population Services International (PSI) in collaboration with the Society for Family Health (SFH) in Nigeria, design partner IDEO.org, and the Centre for the Developing Adolescent at the University of California, collectively referred to here as the ‘implementers’. The implementers were all involved in the design process. PSI and SFH are responsible for the larger scale operationalising of the designed interventions. A360 is co-funded by the Bill & Melinda Gates Foundation and the Children’s Investment Fund Foundation.

**Figure 1.**
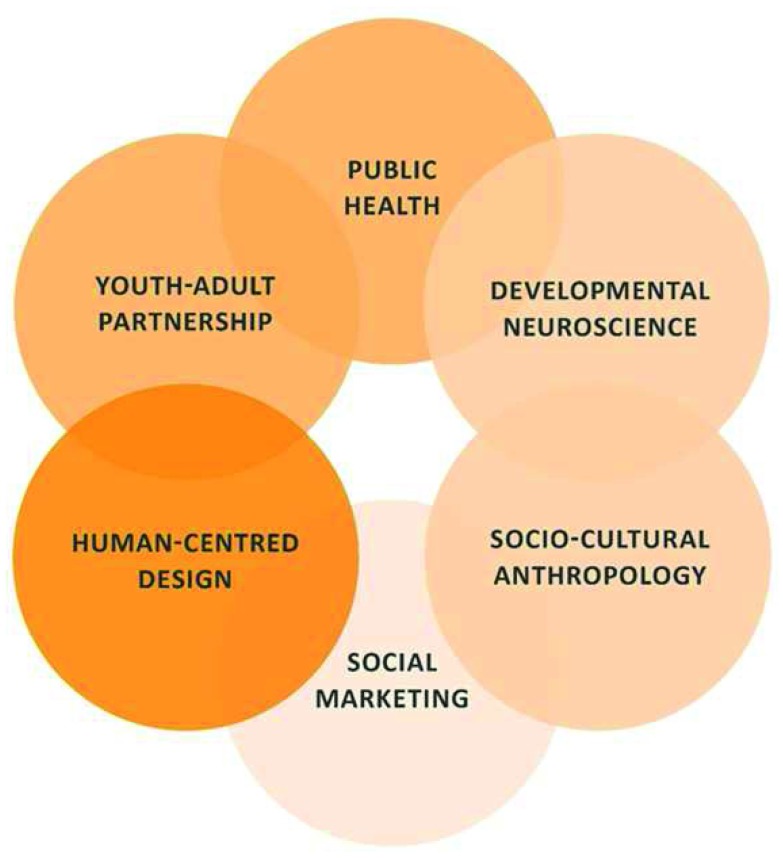
The A360 approach.

A360 set out to take a transdisciplinary approach to intervention development which involves the different discipline ‘lenses’ working jointly to create new innovations that integrate and move beyond discipline-specific approaches to address a common problem ( Figure 1).

A360 aims to create context-specific, youth-driven solutions that respond to the needs of young people. Through an iterative design process, unique solutions were created in each of the A360 countries. In Ethiopia, Smart Start uses financial planning as an entry point to discuss contraception with newly married couples ^17^. In southern Nigeria, 9JA girls provides physical safe spaces (branded spaces in public health clinics) for unmarried girls ^18^, and in northern Nigeria, Matasa Matan Arewa targets married girls and their husbands with counselling and contraceptive services using maternal and child health as an entry point ^19^. In Tanzania ‘Kuwa Mjanja’ (be smart) delivers life and entrepreneurial skills training alongside opt-out counselling sessions and on-site contraceptive service provision to both married and unmarried adolescent girls ^20^.

## External evaluation

**Figure 2.**
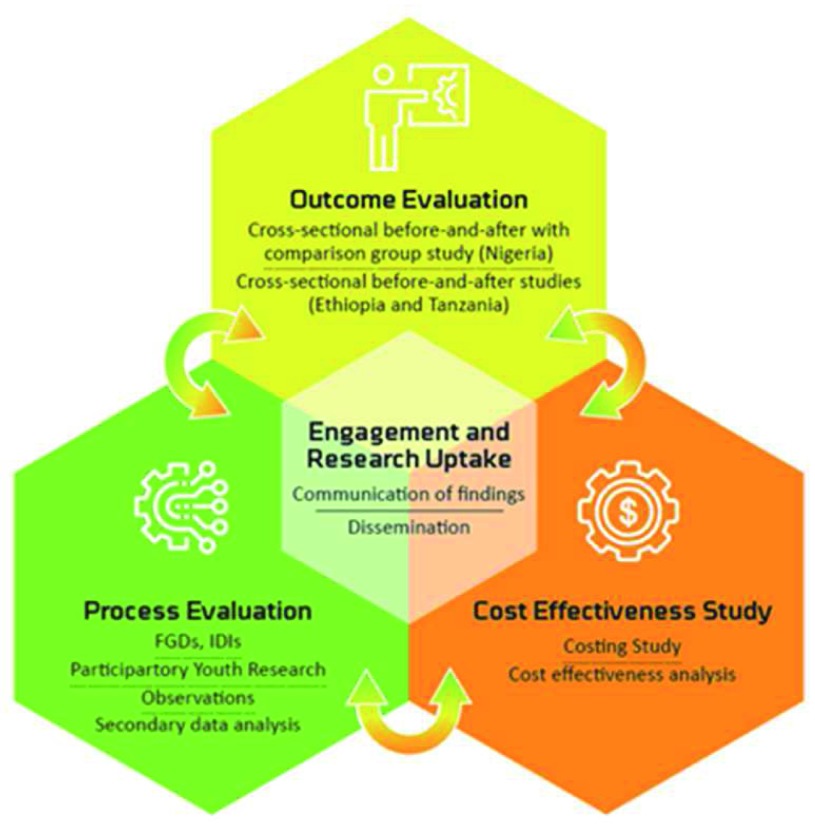
A360 multi-component external evaluation.

The independent external evaluation aims to understand whether the A360 approach leads to improved reproductive health outcomes; generate evidence about A360’s cost-effectiveness in relation to other programming approaches; and capture how A360 is implemented in different contexts, and the experience of the participating young people and communities. It has four components ( Figure 2) ^21^:

1. Outcome evaluation which has a quasi-experimental design with before-after cross-sectional surveys in all four settings (Oromia, Ethiopia; Mwanza, Tanzania; Ogun and Nasarawa, Nigeria) and a comparison group in the two Nigerian settings ^22^.
2. Process evaluation incorporating both traditional qualitative methodologies and youth participatory research ^23^.
3. Costing study to understand the drivers of cost and a more extensive cost-effectiveness study within the outcome evaluation study geographies.
4. Engagement and research uptake.

## HCD process and timeline

HCD takes a phased approach to intervention development. In the ‘inspiration phase’ the designers immerse themselves with girls and their influencers to understand the issues. In the ideation phase they attempt to make sense of what has been learnt, identify opportunities for design and conduct an iterative cycle of prototyping of possible solutions and strategies. The most promising prototypes are then piloted. In the case of A360, implementation at scale involved a further ‘optimisation’ period where the selected solutions were further modified to maximise scalability and affordability. In A360, the inspiration and ideation phases took place over approximately 12 months (Sept 16–Aug 17) with piloting for a further 3–4 months (September–December 17). Optimisation and scale-up started towards the end of 2017/early 2018. Among other things, the length of the intervention design period presented some challenges for the evaluation, which we explore in the sections that follow.

## Outcome evaluation

**Figure 3.**
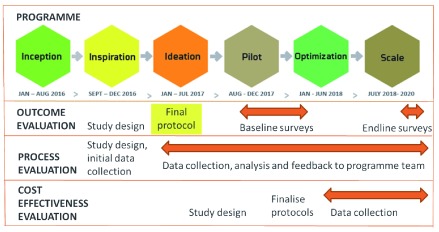
Timeline of A360 intervention development and implementation and the external evaluation.

The outcome evaluation aimed to measure the impact of A360 on the voluntary use of modern contraceptives among adolescent girls aged 15–19 years in the study geographies. The outcome evaluation was designed when the implementers were at the ‘inspiration phase’ with final study protocols submitted for ethical approval when prototyping was still ongoing and before the intervention had been finalised ( Figure 3). This outcome evaluation timeline was necessary in order to conduct baseline surveys in 2017 prior to scale-up of the finalised interventions in the four study settings. The implications of the concurrent intervention development and evaluation design were that key pieces of information about the intervention components and the theory of change were unclear and changed over the protocol development period. Detailed information on the intervention were needed to inform the setting for baseline surveys and the kinds of questions to be asked during the surveys. The outcome evaluation study design was finalised before the interventions themselves so there remained a risk that the evaluation was not optimal.

A key consideration for the baseline survey design was who would be targeted with the A360 intervention in terms of geographical location and demographic characteristics. Early on in the intervention design process it became clear that A360 would be targeted towards only married or unmarried adolescent girls in some locations though uncertainty remained through early phases of the design process. For example, in Northern Nigeria, the outcome evaluation focused on married adolescent girls as the initial intention of A360 was to target only those who were married, however, 9ja Girls, the intervention that was designed in Southern Nigeria to target unmarried adolescent girls, was eventually also implemented in Northern Nigeria. In Ethiopia, initial HCD insights pointed towards a focus on unmarried adolescents in school but other programming pressures including donor preferences led to a later shift in focus to married rural women. The outcome evaluation was designed to target all married 15–19-year-old girls, but the final intervention focused on ‘newly married’ adolescent girls. While some change in target population might be expected with non-HCD programmatic interventions, the HCD process provided the implementers with more freedom and confidence to make additional changes. It was challenging to determine the geographies that would receive A360 as the implementers were in the middle of an intense phase of solution prototyping and were not yet thinking about larger scale implementation of A360.

The level of implementation of the intervention was also relevant for study design, e.g. would A360 be implemented at health facilities, in schools or in the community? Given the uncertainty as to how A360 would be implemented, we opted for a community-based survey, which turned out to be appropriate for the final A360 interventions. However, if A360 had been implemented primarily in health facilities or schools then evaluating the intervention at the broader community level might not have been the most efficient study design.

The uncertainties in key study design parameters meant that we had to develop multiple study design scenarios which were repeatedly revised as new information came to light. For example, initially the intervention was to have distinct implementation phases as it was rolled out across Nigeria and we explored the idea of conducting a stepped-wedge trial. Following further discussions with the implementers, it became clear that there was no certainty that roll-out would be in a phased manner as they did not know yet what the intervention would comprise. In Tanzania, we explored whether a regression discontinuity design might be possible if the intervention were implemented in one or several of the existing demographic surveillance sites, however, given the uncertainty around the nature of the intervention, the implementers were reluctant to commit to implementing in those areas. This prolonged study design process entailed some added costs and was at times frustrating for all involved.

## Process evaluation

The process evaluation was aligned to the HCD-driven phases of the design process for A360 and had the primary objective of presenting a descriptive and analytical account of how the implementation of A360 has played out in relation to its Theory of Change ^23^. The process evaluation attempted to understand both the intervention development process and intervention implementation. During intervention development the process evaluation team faced challenges as the fast-paced, highly iterative HCD process meant that the ‘design energy’ i.e. how decisions were made at key points in the design process, often went undocumented ^4^. This challenge was also noted in another evaluation of a HCD design process ^8^. As a result, the process evaluation team needed to be flexible in order to align closely with the work plan of the implementers and methodologies such as direct participant observation were key to capturing the depth of the HCD process.

The potential for research fatigue was observed among target community members as their views were solicited by both the implementers designing the intervention and the process evaluation team interested in understanding the HCD process from the participants’ point of view. The process evaluation team, therefore, needed to balance the importance of capturing the views of community members with the potential for research fatigue.

During the design of the process evaluation, the intention was that the findings would feed into and inform the intervention design at key moments. However, there was limited uptake of process evaluation findings by implementers. For example, the process evaluation highlighted the need for the programme to do more to address broader community and social norms, but this finding had a limited impact on intervention design. Poor uptake of findings was partly a result of the fast-paced nature of A360 and the resultant demands on the implementers’ time which did not allow them sufficient time to pause and reflect on the process evaluation findings. Country implementing teams differed in how they engaged with external recommendations, with some teams receptive and willing to listen and adjust, while others were more protective of their ‘solutions’. Uptake of the process evaluation findings improved when the evaluation team introduced participatory action research activities which focused on operational questions that were important for the country teams (e.g. health care provider attitudes in Ethiopia ^24^ and Nigeria ^25^).

## Cost-effectiveness evaluation

The cost-effectiveness study faced similar challenges as the outcome evaluation in terms of complicated and delayed development of the cost-effectiveness study protocols. Although all costing exercises face some unknowns about the intervention to be costed, the flexible and iterative HCD process increased the number of unknowns. Furthermore, because proponents hypothesized that it was the design process itself that would be the key factor in producing an effective (and cost-effective) intervention, an important challenge was both measuring the total design cost and isolating the cost of HCD specifically as HCD activities of the design process were tightly intertwined with the other A360 ‘lenses’. In addition, because A360 was creating a new trans-disciplinary approach, there was also interest in separating out the costs associated with the ‘creation’ of the A360 approach from the costs associated with implementing and scaling A360 in countries. Interviews were held with intervention staff in order to get a sense of the distribution of activities (and associated costs) across the study ‘lenses’ and between efforts to create versus implement A360. The implementing agencies planned continuous changes or tweaks to the intervention once it was up and running and so more frequent cost data draws were required during the scale-up period to capture how those changes might affect the “production process” and associated costs. 

## Lessons learnt

Robust evaluation of a new and promising approach such as HCD is warranted yet challenging. At the start of the programme, there was a clear methodologic gap between the evaluation team who had a background in public health and the social sciences, and the design team at IDEO who were leading the HCD process. However, PSI and the other implementers, who were working closely with IDEO, also had a background in public health and the social sciences and helped bridge this gap. Also, the evaluation team had been thoroughly briefed about the HCD methodologies that would be used but they had only limited practical experience evaluating programmes incorporating human-centered design.

Some of the challenges faced are not unique to interventions designed with HCD and will be recognisable by those who have conducted interdisciplinary research and/or led evaluations of programmatic interventions. In comparison to research-led studies, evaluation of programmatic interventions is associated with a reduced level of control and increased uncertainty. A challenge for evaluators is to find ways to deal with this uncertainty while still retaining scientific rigor. An additional long-standing challenge, not specific to HCD, is to convince implementers that rigorous evaluation, while at times intrusive, will improve programme design and implementation. 

We have the following recommendations for others who would like to evaluate programmes incorporating HCD:

1. Evaluators and implementers/designers should take time to familiarise themselves with the methodologies used by the different disciplines and have open discussions about the potential challenges of interdisciplinary research, and how they will be addressed/mitigated against.
2. Implementers should allow adequate time to participate in the process evaluation, as well as work with the process evaluation team to ensure that findings are timed to feed into key decisions.
3. The process evaluation team should maximise secondary analysis of data collected by the implementers, and joint data collection could be considered where additional data collection is needed and participant research fatigue is anticipated.
4. Like HCD, an iterative and adaptive process evaluation approach is required. In A360, the process evaluation paused after the pilot phase and our team worked with the A360 implementers and donors to develop evaluation questions that reflected the solutions that were being developed, reflecting an iterated A360 Theory of Change.
5. Methodologies such as participant action research may identify process evaluation questions that are more relevant for the implementers. For the evaluators direct observations can be instrumental to capturing the fast-paced, highly-iterative HCD process, and to understand the ‘design energy’ i.e. how decisions were made at key points in the design process.
6. To avoid a time-consuming and resource-intensive design process, future HCD-based initiatives should consider a phased evaluation approach:
  • Conduct process evaluation during the HCD inspiration, ideation, and pilot phases.
  • Wait until the implementers have a better understanding of the emerging programme and have finalised the target geographies, target population, and intended outcomes before planning an outcome evaluation and cost-effectiveness study.
  • During the implementation phase, conduct a comprehensive process evaluation that can capture whether, how, and why the intervention changed during implementation.

The advantages of a phased approach need to be balanced against the disadvantages of lengthening of the time between implementation and the availability of evaluation findings.

## Data availability

No data are associated with this article.

## References

[1] Bazzano AN Martin J Hicks E: Human-centred design in global health: A scoping review of applications and contexts. *PLoS One.* 2017;12(11):e0186744. 10.1371/journal.pone.0186744 29091935 PMC5665524

[2] Kolko J: Design Thinking Comes of Age. *Harvard Business Review.* 2015 Reference Source

[3] IDEO.org: The Field Guide to Human-centered design. IDEO.org/Design Kit;2015 Reference Source

[4] Tolley EB: Traditional Socio-behavioral research and human-centered design: Similarities, Unique Contributions and Synergies.2017 Reference Source

[5] Evaluation in health and wellbeing.2018; (Accessed 08 Mar 19, 2019). Reference Source

[6] Webster J Exley J Copestake J: Timely evaluation in international development. *J Dev Effect.* 2018;10(4):482–508. 10.1080/19439342.2018.1543345

[7] LaFond A Davis N: Evaluating the Influence of Human-Centered Design on Health Programs.The Pump;2017 Reference Source

[8] Itad: Evaluation of the Hewlett Foundation's Strategy to Apply Human-Centered Design to improve Family Planning and Reproductive Health Services in Sub-Saharan Africa. Final Report.2017 Reference Source

[9] Rogers P: Does evaluation need to be done differently to support adaptive management? BetterEvaluation;2017 Reference Source

[10] Simister N: How Adaptive Management is challenging the monitoring and evaluation of complex programmes.intrac for civil society;2018 Reference Source

[11] Valters C Cummings C Nixon H: Putting learning at the centre. Adaptive development programming in practice.London, UK: ODI;2016 Reference Source

[12] Barry D Kimble LE Nambiar B: A framework for learning about improvement: embedded implementation and evaluation design to optimize learning. *Int J Qual Health Care.* 2018;30(suppl_1):10–4. 10.1093/intqhc/mzy008 29873794 PMC5909667

[13] Øvretveit J Gustafson D: Evaluation of quality improvement programmes. *Qual Saf Health Care.* 2002;11(3):270–5. 10.1136/qhc.11.3.270 12486994 PMC1743631

[14] Waiswa P Manzi F Mbaruku G: Effects of the EQUIP quasi-experimental study testing a collaborative quality improvement approach for maternal and newborn health care in Tanzania and Uganda. *Implement Sci.* 2017;12(1):89. 10.1186/s13012-017-0604-x 28720114 PMC5516352

[15] Birdthistle I Schaffnit SB Kwaro D: Evaluating the impact of the DREAMS partnership to reduce HIV incidence among adolescent girls and young women in four settings: a study protocol. *BMC Public Health.* 2018;18(1):912. 10.1186/s12889-018-5789-7 30045711 PMC6060450

[16] Chandrasekaran P Dallabetta G Loo V: Evaluation design for large-scale HIV prevention programmes: the case of Avahan, the India AIDS initiative. *AIDS.* 2008;22 Suppl 5:S1–15. 10.1097/01.aids.0000343760.70078.89 19098469

[17] A360: Country Solutions- Ethiopia.2019; (Accessed 28 Mar 19). Reference Source

[18] A360: Country Solutions- Southern Nigeria.2019; (Accessed 28 Mar 19). Reference Source

[19] A360: Country Solutions- Northern Nigeria.2019; (Accessed 28 Mar 19). Reference Source

[20] A360: Country Solutions- Tanzania.2019; (Accessed 28 Mar 19). Reference Source

[21] Adolescents 360 external evaluation.2019; (Accessed 28 Mar 19). Reference Source

[22] Atchison CJ Mulhern E Kapiga S: Evaluating the impact of an intervention to increase uptake of modern contraceptives among adolescent girls (15-19 years) in Nigeria, Ethiopia and Tanzania: the Adolescents 360 quasi-experimental study protocol. *BMJ Open.* 2018;8(5):e021834. 10.1136/bmjopen-2018-021834 29858422 PMC5988138

[23] Mulhern E Appleford G: Adolescents 360 Evaluation: Process evaluation methodology. Itad;2018 Reference Source

[24] Itad: Adolescents 360 Evaluation: How might we better meet the needs of adolescent couples with contraceptive counseling and services through Ethiopia's Health Extension Program?2018 Reference Source

[25] Itad: Adolescents 360 Evaluation: What do service providers think about contraceptive service provision to 15–19 year old girls in Nigeria?2018 Reference Source

