# Supplementary material for: Challenges and opportunities in evaluating programmes incorporating human-centred design: lessons learnt from the evaluation of Adolescents 360
Source: Gates Open Res. 2019 Sep 25;3:1472. Originally published 2019 May 24. [Version 2] doi: 10.12688/gatesopenres.12998.2 (PMC6635668; doi:10.12688/gatesopenres.12998.2)
Supplement: Peer review history

# Peer review: Reviewer response for version 2

**DOI:** 10.21956/gatesopenres.14206.r27930

**Published:** 2019-10-02

**Authors:** Melissa Gilliam

**Affiliations:** 1 The Centre for Interdisciplinary Inquiry & Innovation in Sexual Health and Productive Health, University of Chicago, Chicago, IL, USA

The authors have been very responsive in their revisions and I have no further concerns. Again, this article makes a valuable contribution to the literature.

I confirm that I have read this submission and believe that I have an appropriate level of expertise to confirm that it is of an acceptable scientific standard.

# Peer review: Reviewer response for version 1

**DOI:** 10.21956/gatesopenres.14106.r27351

**Published:** 2019-07-15

**Authors:** Kristien Michielsen

**Affiliations:** 1 International Centre for Reproductive Health (ICRH), Ghent University, Ghent, Belgium

The article presents lessons learnt from an external evaluation of the A360 programme in three African countries. A360 uses the human-centred design and hence applied three types of evaluations to evaluate it: whether the programme leads to improved reproductive health outcomes (outcome evaluation), capture how it is implemented (process evaluation) and study its cost-effectiveness. Additionally engagement and research uptake.

 The article honestly presents the known tension between effective implementation of a context-specific and flexible interventions versus an experimental evaluation design. It is refreshing to read an honest reflection on this, and as someone who has done similar evaluations, it clearly recognize the challenges. However, it would be even better if more specific recommendations would have been given on how to actually deal with this tension.

 Limited uptake of the process evaluation results by the implementers: it would have been interesting to understand why some implementers were eager to take on recommendations and others were not, and how this affected the effectiveness.

 Cost-effectiveness: since the effectiveness study was somewhat compromised, it is difficult to link it to the costs. It feels as if the cost-effectiveness study mainly mapped the costs, without linking it to the effectiveness.

 Reflections:

- The lessons learnt are important, but stem from common sense and are not new. While it is often clear what should be done, the challenge remains on how to do it: how to convince donors and implementers of the importance of proper evaluations and its consequences for the programme design and implementation.
- Cost-effectiveness of the evaluation: a lot of time and energy was given to do three types of evaluations. However, it seems as it there value was not optimal. It may be interesting to reflect on the cost-effectiveness of the evaluation itself.
- How were the different evaluations lied together? They seem to be presented quite separately, while for a clear understanding of the programme, they should be tied together.
- How is the evaluation approach chosen better/worse than e.g. realist evaluation, which seems quite appropriate for this type of intervention/programme.

I confirm that I have read this submission and believe that I have an appropriate level of expertise to confirm that it is of an acceptable scientific standard.

# Response

**Published:** 2019-09-12

**Authors:** Aoife Doyle

The article presents lessons learnt from an external evaluation of the A360 programme in three African countries. A360 uses the human-centred design and hence applied three types of evaluations to evaluate it: whether the programme leads to improved reproductive health outcomes (outcome evaluation), capture how it is implemented (process evaluation) and study its cost-effectiveness. Additionally engagement and research uptake.

The article honestly presents the known tension between effective implementation of a context-specific and flexible interventions versus an experimental evaluation design. It is refreshing to read an honest reflection on this, and as someone who has done similar evaluations, it clearly recognize the challenges. However, it would be even better if more specific recommendations would have been given on how to actually deal with this tension.

***Response: Thanks. We have added a new recommendation (1) and revised recommendation (4) to suggest that evaluators and implementers need to invest time upfront to become familiar with each other’s methodologies and then to have more open discussions prior to the study on the potential challenges that they may face in the conduct of interdisciplinary research.***

Limited uptake of the process evaluation results by the implementers: it would have been interesting to understand why some implementers were eager to take on recommendations and others were not, and how this affected the effectiveness.

***Response: Yes, we agree that this is interesting. This issue is being explored through ongoing process evaluation and the findings will be made available at a later date.***

Cost-effectiveness: since the effectiveness study was somewhat compromised, it is difficult to link it to the costs. It feels as if the cost-effectiveness study mainly mapped the costs, without linking it to the effectiveness.

***Response: The cost-effectiveness and outcome evaluation studies are still ongoing. When the effectiveness results are available following endline surveys in 2020, the findings will be used in the cost-effectiveness analysis.***

Reflections:

- The lessons learnt are important, but stem from common sense and are not new. While it is often clear what should be done, the challenge remains on how to do it: how to convince donors and implementers of the importance of proper evaluations and its consequences for the programme design and implementation.

***Response: In recognition of this issue we have added the following sentence:***

***‘An additional long-standing challenge, not specific to HCD, is to convince implementers that rigorous evaluation, while at times intrusive, will improve programme design and implementation.’***

- Cost-effectiveness of the evaluation: a lot of time and energy was given to do three types of evaluations. However, it seems as it there value was not optimal. It may be interesting to reflect on the cost-effectiveness of the evaluation itself.

***Response: The three evaluations are presented separately as they represent separate streams of the evaluation work but are very integrated and will be presented and interpreted together at the end of the project. This is an ongoing evaluation and so it is too early to reflect on the cost-effectiveness of the evaluation itself.***

- How were the different evaluations lied together? They seem to be presented quite separately, while for a clear understanding of the programme, they should be tied together.

**See previous response.**

- How is the evaluation approach chosen better/worse than e.g. realist evaluation, which seems quite appropriate for this type of intervention/programme.

***Response: Our evaluation approach focuses on several key process, outcome and cost measures so we are to some extent capturing the context- mechanism-outcome configurations that are described in the realist evaluation literature(1, 2). However, the outcome evaluation is powered to look at the average impact of the intervention across the total population (Does it work?) and so we will have limited power to look at intervention impact in different sub-groups (What works for whom and where?), which is an essential part of a realist evaluation. It is possible that the iterative nature of HCD would necessitate more frequent revisits of the programme theory and CMO statements and would hence be relatively more expensive that a realist evaluation of a non-HCD programme.***

***1.            Pawson R, Tilley N. Realistic Evaluation. London: Sage; 1997.***

***2.            Westhorp G. Realistic Impact Evaluation. An Introduction.: ODI; 2014.***

# Peer review: Reviewer response for version 1

**DOI:** 10.21956/gatesopenres.14106.r27411

**Published:** 2019-07-15

**Authors:** Melissa Gilliam

**Affiliations:** 1 The Centre for Interdisciplinary Inquiry & Innovation in Sexual Health and Productive Health, University of Chicago, Chicago, IL, USA

In this article, the authors discuss challenges and opportunities associated with evaluating HCD interventions. In this case, the intervention was associated with A360.

 Overall, the honesty of the authors is appreciated as rarely does one get to hear about problems that occurred during project implementation. Reporting on successes and failures is important as it provides an opportunity for others to learn. Nevertheless, the descriptions of the challenges that arose were a little cryptic and it was not always clear.

 I was also disappointed that the authors did not reach another logical conclusion which is that there was a methodologic gap between the public health researchers/evaluators and designers which lead to the challenges. Conducting interdisciplinary research can be challenging and requires each team to be very familiar with the other’s methods and processes so they can carefully design the collaboration. For example, when the study populations grow frustrated with the multiple inquiries it suggests that the data collection was not harmonized across teams. Similarly, the breakdown in communication emerges. These are the classic challenges of interdisciplinary research and may not only apply to HCD. The authors might discuss how they prepared to work with human centered designers or vice versa. What type of training took place and how familiar were the evaluators with the design process or were the designers with public health methods?

 Specific comments are as follows:

1. It is interesting that the intervention design phase appears long. If one were to compare it to drug development or another multiphase intervention development process, then two years would be quite short.
2. It is not clear why baseline data were collected prior to developing the intervention. How does collecting data in the four settings, the explanation provided here, constrain when data are collected?
3. In looking at the diagram of the timeline, it seems that the baseline survey could have been moved up to July 2018 once all decisions were made regarding what the study would be about.
4. The process evaluation is very interesting as is the objective of including insights into the final intervention. However, I was not clear what was meant by including PAR improved uptake. Is the author saying that they collected data using PAR that was of interest to the designers? If so, that would suggest that a recommendation might be that each team needs more familiarity with the array of methods available for process research prior to starting the program so data collection could be designed in collaboration.
5. Cost effectiveness - it is not clear what the authors mean by a layer of uncertainty created by HCD.
6. Further, it is not clear why the cost of HCD was being assessed rather than the cost of the intervention.
7. Lessons learnt, item 5 is very important as is anticipating issues of interdisciplinary research from the beginning.

I confirm that I have read this submission and believe that I have an appropriate level of expertise to confirm that it is of an acceptable scientific standard, however I have significant reservations, as outlined above.

# Response

**Published:** 2019-09-12

**Authors:** Aoife Doyle

In this article, the authors discuss challenges and opportunities associated with evaluating HCD interventions. In this case, the intervention was associated with A360.

Overall, the honesty of the authors is appreciated as rarely does one get to hear about problems that occurred during project implementation. Reporting on successes and failures is important as it provides an opportunity for others to learn. Nevertheless, the descriptions of the challenges that arose were a little cryptic and it was not always clear.

I was also disappointed that the authors did not reach another logical conclusion which is that there was a methodologic gap between the public health researchers/evaluators and designers which lead to the challenges. Conducting interdisciplinary research can be challenging and requires each team to be very familiar with the other’s methods and processes so they can carefully design the collaboration. For example, when the study populations grow frustrated with the multiple inquiries it suggests that the data collection was not harmonized across teams. Similarly, the breakdown in communication emerges. These are the classic challenges of interdisciplinary research and may not only apply to HCD. The authors might discuss how they prepared to work with human centered designers or vice versa. What type of training took place and how familiar were the evaluators with the design process or were the designers with public health methods?

***Response: Thanks for highlighting this omission. We have added the following text to the ‘Lessons learnt’:***

******

***‘At the start of the programme, there was a clear methodologic gap between the evaluation team who had a background in public health and the social sciences, and the design team at IDEO who were leading the HCD process. However, PSI and the other implementers, who were working closely with IDEO had a background in public health and the social sciences. The evaluation team had been thoroughly briefed about the HCD methodologies that would be used but they had only limited practical experience evaluating programmes incorporating human-centered design. Some of the challenges faced are not unique to interventions designed with HCD and will be recognisable by those who have conducted interdisciplinary research and/or led evaluations of programmatic interventions.’***

Specific comments are as follows:

- It is interesting that the intervention design phase appears long. If one were to compare it to drug development or another multiphase intervention development process, then two years would be quite short.

***Response: We agree that intervention development can often take a long time. We have edited the text to remove the word ‘lengthy’ and instead state ‘the length of the intervention design period’.  ***

- It is not clear why baseline data were collected prior to developing the intervention. How does collecting data in the four settings, the explanation provided here, constrain when data are collected?

***Response: The baseline surveys needed to be conducted prior to the scale-up of the interventions i.e. so that we had baseline values for our primary and secondary outcomes. In the sentence in question, we have moved the words ‘prior to scale-up ‘ so that they appear before ‘in the four study settings’. We hope that this is now clearer. ***

- In looking at the diagram of the timeline, it seems that the baseline survey could have been moved up to July 2018 once all decisions were made regarding what the study would be about.

***Response: In hindsight, yes this would have made sense but the scale-up of the interventions were originally due to start in January 2018. The optimization phase was introduced as an additional phase only towards the end of 2017 and the extension of the evaluation period into 2020 (previously was due to end in 2019) was agreed in 2018.***

- The process evaluation is very interesting as is the objective of including insights into the final intervention. However, I was not clear what was meant by including PAR improved uptake. Is the author saying that they collected data using PAR that was of interest to the designers? If so, that would suggest that a recommendation might be that each team needs more familiarity with the array of methods available for process research prior to starting the program so data collection could be designed in collaboration.

***Response: Thanks, yes this is a good observation. We have added this to recommendation 4:***

***4. For the process evaluation, methodologies such as participant action research may identify questions that are more relevant for the implementers, and for the evaluators direct observations can be instrumental to capturing the fast-paced, highly-iterative HCD process, and to understand the ‘design energy’ i.e. how decisions were made at key points in the design process.***

- Cost effectiveness - it is not clear what the authors mean by a layer of uncertainty created by HCD.

***Response: We have replaced this phrase with ‘the flexible and iterative HCD process increased the number of unknowns’.***

- Further, it is not clear why the cost of HCD was being assessed rather than the cost of the intervention.

***Response: The cost of the design process is being collected as is the cost of implementing the intervention itself. Within the design costs, the cost of HCD is being estimated. A360 is based on the idea that using an integrated HCD process to design the interventions will result in more impactful interventions. Therefore, the cost-effectiveness evaluation is interested in capturing the design costs as well as the intervention cost in order to benchmark against the costs and cost-effectiveness of a more traditional design process.***

- Lessons learnt, item 5 is very important as is anticipating issues of interdisciplinary research from the beginning.

***Response: we have added an additional recommendation on the issue of interdisciplinary research:***

***‘1. Evaluators and implementers/designers should take time to familiarise themselves with the methodologies used by the different disciplines and have open discussions about the potential challenges of interdisciplinary research, and how they will be addressed/mitigated against.’***

# Peer review: Reviewer response for version 1

**DOI:** 10.21956/gatesopenres.14106.r27240

**Published:** 2019-06-26

**Authors:** Isaac Holeman

**Affiliations:** 1 Medic Mobile, Seattle, WA, USA; 2 University of Washington, Seattle, WA, USA

**Overview**

 This letter offers reflections on the authors’ experience evaluating an HCD-led global health program called A360. The letter is timely in that it raises questions that many global health programs have grappled with of late. I particularly appreciate the authors’ frankness as they describe disconnects between design/implementation work and research. On the whole I am very enthusiastic about this letter moving forward in the publication process. I would like to offer a few reflections of my own in the hope of helping the author’s improve their letter and of helping our research community identify better solutions to these deeply important challenges. 

**Major comments**

 This letter seems to focus on the relationship between health research and design practice, and my primary question has to do with how the authors’ experiences and conclusions might have differed if their project had emphasized linking health research and design research. To be clear, I’m not referring to the way that practitioners refer to doing ‘design research’ or ‘user research’ to investigate user experiences and contexts. By design research, I’m referring to the program of empirical study that has been carried out by human-centered design researchers in the fields of design, information systems, computer supported cooperative work (CSCW), human computer interaction, and design anthropology (and beyond) in the last few decades. These fields draw heavily on ethnographic, participatory, and action research methods that are familiar to many process evaluators in global health (no doubt including the authors of this letter). Design researchers in these fields also often draw on domain-specific craft skills that enable visual expression and prototyping, intervention development and implementation, and iteration based on ‘hands-on’ engagement with end users. That is to say, in fields with a more established tradition of design research, the researchers often are designers. For methods of contemporary importance, see e.g., action design research ^1^ and research through design ^2^. For a paper that is older but perhaps more relevant to my comment here, see Spinuzzi’s ^3^ argument that participatory design is a design method, yet also is an action-research method.

 While some of these design-led research fields started with collaborations across disciplines (e.g. ethnographers and computer scientists founded CSCW in the early 1990s), today doctoral students typically learn to integrate design practices and research methods in the conduct of their scholarly endeavours. Integrating design practice with research method thus becomes less a matter of coordinating two teams within an organizational bureaucracy, and something more akin to how an individual ethnographer strives for reflexivity concerning her dual roles as participant and as observer. This seems to contrast with the research described in this letter, in which the evaluations were conducted by researchers who did not consider themselves as ‘doers’ of the design work (if I’m mistaken about this point, I’d welcome being corrected!).

 In light of the example set by human-centered design researchers in CSCW and adjacent fields, my more precise question is this: how might the challenges you encountered be reconsidered as having to do with the all too common gap between researchers and practitioners? In particular I’m thinking of these challenges:

- The frustrating communications that resulted from a prolonged design process.
- Research fatigue among participants, and the importance of jointly collecting data for design and for research so that people need not be interviewed twice.
- The need for evaluators to be present, as participant-observers in design work in order to understand the design-energy and design decisions.
- Limited uptake of findings generated by process evaluators.
- Implementers/designers needing to set aside time to participate in a process evaluation.
- The importance of ensuring that the process evaluation is phased and iterative in a manner that matches the design work.

In my experience, these are common problems/risks wherever researchers try to plug into (but aren’t leading) a process led by practitioners, regardless of the field of practice. Often this is no fault of the practitioners - understanding and accommodating a scientific agenda is often beyond their remit. These gaps can be alleviated to some degree when there is one integrated design team that has the skills, methodological background, and remit to conduct a design process, produce evidence about user experiences to iteratively inform intervention development, and use this evidence to evaluate and write about their process (see e.g., ^4^^,^^5^^,^^6^^,^^7^).

 To be fair, the approach outlined in this letter seems at present to be the norm rather than the exception in global health journals: enlisting a team of researchers, who are not designers, to evaluate design practitioners, who are not researchers. The reason I find this letter so valuable is that it surfaces a set of challenges that may be inherent in this approach, and the authors should be commended for their frankness.

 The authors will need to discern among themselves whether, based on their experience, they would encourage future evaluators of design-led health initiatives to include researchers from disciplines with more established traditions of integrating design research and practice. If this does resonate with your experience, I would offer two sources that you might reference that speak to a way forward for the design for global health community. The ISO standard on human-centered design ^8^ and more recent work on design for global health ^9^ emphasize that human-centered design is a highly multidisciplinary approach to research and practice - it is centrally concerned with the practical task of synthesizing diverse perspectives. This point might be used to argue that involving design researchers alongside the range of other research disciplines outlined in the A360 approach is a way forward for streamlining adoption of your five recommendations.

 As a final point, I would acknowledge that while the tradition of design research with which I am most familiar can well serve the role of process evaluation as it plays out in the global health field, outcomes evaluations and cost-effectiveness studies remain more the domain of public health researchers and health economists. Such studies can be conducted relatively straightforwardly if they focus on the interventions that resulted from a design process. While good enough for most policy purposes, that approach doesn’t address the question of what proportion/aspect of good outcomes we might attribute specifically to the application of human-centered design. Given the complexity of the design process I don’t know the answer here, and while the letter raises the question, the authors’ five recommendations don’t seem to address it either. One could follow the approach taken in evaluating the impact of applying behavior change theories in the design of communication interventions - meta-analyses of many studies with and without HCD. Such an approach would likely surface a further round of questions, unaddressed in this letter, about the extent to which the impacts of design work can/should be attributed to the design practitioner’s degree of expertise, or whether a project was structured to enable the design practice to thrive, versus something integral to human-centered design as a discipline.

**Minor Comments:**

 It might be helpful if you clarified your use of the terms “implementer” and “designer” throughout the paper. Are you referring to implementers of the design work (i.e., designers), or to other implementers who were operationalizing the interventions that the designers designed, or both?

 The reference below to ‘design energy’ is intriguing; I think readers would appreciate slightly more explanation of what this refers to and in what sense the term is dealt with in the cited reference.

 “During intervention development the process evalu- ation team faced challenges as the fast-paced, highly iterative HCD process meant that the decision making process, the ‘design energy’, often went undocumented ^4^.”

 In the statement below, I wasn’t able to follow how starting with a blank slate may have blocked the the design team from drawing on public health evidence. Design researchers and ethnographers typically strive to approach observational fieldwork in a manner that minimizes the influence of preconceived notions, but this does not prevent them from drawing on relevant theories or evidence during the process of analysis. Was disregarding public health evidence explicitly described as typical of the design process (a point I would disagree with in general, but that may well have been true to your experience), or might it have just been another researcher to practitioner disconnect?

 “Designers add value to public health as they take a different approach when address- ing an issue, however, in the case of A360, a preference to work from a blank slate free of the constraints of public health evidence may have impacted on an ability to respond to the PE findings.”

**Typos**

 “However, the iterative and flexible nature of HCD potentially poses some unique chal- lenges for evaluation ^1,3,7^.[ **remove period**] and examples of evaluations of HCD public health interventions have so far been limited ^7,8^.”

 “In the ‘inspiration phase’ the designers immerse themselves with girls and **her their** influencers to understand the issues.”

 “The OE study design was finalised before the interventions themselves so there remained a risk that the evaluation was not optimal.” I think this refers to Outcome Evaluation study design; it would be helpful if you could clarify on first use of the acronym.

 “distinct imple- mentation phases as it was rolled **out** across Nigeria”

 “because A360 was creating a new trans-disciplin **ary** approach”

 “more frequent cost data draws were required during the scale-up period to capture how those change **s** might affect the “production process” and associated costs.”

I confirm that I have read this submission and believe that I have an appropriate level of expertise to confirm that it is of an acceptable scientific standard.

## References

[1] : Action Design Research. Management Information Systems Quarterly.2011;35(1) :37-56

[2] : Research Through Design as a Method for Interaction Design Research in HCI. *CHI*.2007; 10.1145/1240624.1240704 493-502 10.1145/1240624.1240704

[3] : The methodology of participatory design. *Technical Communication*.2005;52(2) :163-174

[4] : Engaging Pregnant Women in Kenya with a Hybrid Computer-Human SMS Communication System.2015; 10.1145/2702123.2702124 1429-1438 10.1145/2702123.2702124

[5] : Adapting Collaborative Radiological Practice to Low- Resource Environments.2012;1-10

[6] : Projecting health.2015; 10.1145/2737856.2738023 1-10 10.1145/2737856.2738023

[7] : Improving the quality of point of care diagnostics with real-time machine learning in low literacy LMIC settings. *Proceedings of the 1st ACM SIGCAS Conference on Computing and Sustainable Societies 2018*.2018;2

[8] Human-centered design for interactive systems. *International Organization for Standardization*.2010;1-40

[9] : Human-Centered Design for Global Health Equity. *Information Technology for Development*.Forthcoming; 10.17605/OSF.IO/7ZTCY 10.17605/OSF.IO/7ZTCY PMC748492132982007

# Response

**Published:** 2019-09-12

**Authors:** Aoife Doyle

**Overview**

This letter offers reflections on the authors’ experience evaluating an HCD-led global health program called A360. The letter is timely in that it raises questions that many global health programs have grappled with of late. I particularly appreciate the authors’ frankness as they describe disconnects between design/implementation work and research. On the whole I am very enthusiastic about this letter moving forward in the publication process. I would like to offer a few reflections of my own in the hope of helping the author’s improve their letter and of helping our research community identify better solutions to these deeply important challenges. 

**Major comments**

This letter seems to focus on the relationship between health research and design practice, and my primary question has to do with how the authors’ experiences and conclusions might have differed if their project had emphasized linking health research and design research. To be clear, I’m not referring to the way that practitioners refer to doing ‘design research’ or ‘user research’ to investigate user experiences and contexts. By design research, I’m referring to the program of empirical study that has been carried out by human-centered design researchers in the fields of design, information systems, computer supported cooperative work (CSCW), human computer interaction, and design anthropology (and beyond) in the last few decades. These fields draw heavily on ethnographic, participatory, and action research methods that are familiar to many process evaluators in global health (no doubt including the authors of this letter). Design researchers in these fields also often draw on domain-specific craft skills that enable visual expression and prototyping, intervention development and implementation, and iteration based on ‘hands-on’ engagement with end users. That is to say, in fields with a more established tradition of design research, the researchers often are designers. For methods of contemporary importance, see e.g., action design research ^1^ and research through design ^2^. For a paper that is older but perhaps more relevant to my comment here, see Spinuzzi’s ^3^argument that participatory design is a design method, yet also is an action-research method.

While some of these design-led research fields started with collaborations across disciplines (e.g. ethnographers and computer scientists founded CSCW in the early 1990s), today doctoral students typically learn to integrate design practices and research methods in the conduct of their scholarly endeavours. Integrating design practice with research method thus becomes less a matter of coordinating two teams within an organizational bureaucracy, and something more akin to how an individual ethnographer strives for reflexivity concerning her dual roles as participant and as observer. This seems to contrast with the research described in this letter, in which the evaluations were conducted by researchers who did not consider themselves as ‘doers’ of the design work (if I’m mistaken about this point, I’d welcome being corrected!).

In light of the example set by human-centered design researchers in CSCW and adjacent fields, my more precise question is this: how might the challenges you encountered be reconsidered as having to do with the all too common gap between researchers and practitioners? In particular I’m thinking of these challenges:

- The frustrating communications that resulted from a prolonged design process.
- Research fatigue among participants, and the importance of jointly collecting data for design and for research so that people need not be interviewed twice.
- The need for evaluators to be present, as participant-observers in design work in order to understand the design-energy and design decisions.
- Limited uptake of findings generated by process evaluators.
- Implementers/designers needing to set aside time to participate in a process evaluation.
- The importance of ensuring that the process evaluation is phased and iterative in a manner that matches the design work.

In my experience, these are common problems/risks wherever researchers try to plug into (but aren’t leading) a process led by practitioners, regardless of the field of practice. Often this is no fault of the practitioners - understanding and accommodating a scientific agenda is often beyond their remit. These gaps can be alleviated to some degree when there is one integrated design team that has the skills, methodological background, and remit to conduct a design process, produce evidence about user experiences to iteratively inform intervention development, and use this evidence to evaluate and write about their process (see e.g., ^4^ ^,^ ^5^ ^,^ ^6^ ^,^ ^7^).

***Response: Thanks for these observations. As is common practice in the field of public health, we are engaged by the donors as external evaluators of this programme and so we deliberately had no role in the design process. It is true that many of the challenges might not have been faced if we had been integrated in the implementing/design team but this would have compromised our ability to remain independent.***

******

***In line with the references that you cite here, the implementers are conducting their own monitoring and evaluation to examine process level indicators e.g. the number of girls reached with their services. The external evaluation, conducted by researchers who specialise in evaluation, uses more complex evaluation methodologies to evaluate the impact of the intervention on proximate and also more distal outcomes e.g. population level use of modern contraceptives. The use of design research in the field of public health is relatively new and as external evaluators gain experience of design research they may need to consider alternative methodologies or ways of working with implementers. In response to suggestions from the other reviewers we have edits the recommendations to suggest improved communication between the external evaluators and implementers.***

To be fair, the approach outlined in this letter seems at present to be the norm rather than the exception in global health journals: enlisting a team of researchers, who are not designers, to evaluate design practitioners, who are not researchers. The reason I find this letter so valuable is that it surfaces a set of challenges that may be inherent in this approach, and the authors should be commended for their frankness.

The authors will need to discern among themselves whether, based on their experience, they would encourage future evaluators of design-led health initiatives to include researchers from disciplines with more established traditions of integrating design research and practice. If this does resonate with your experience, I would offer two sources that you might reference that speak to a way forward for the design for global health community. The ISO standard on human-centered design ^8^and more recent work on design for global health ^9^ emphasize that human-centered design is a highly multidisciplinary approach to research and practice - it is centrally concerned with the practical task of synthesizing diverse perspectives. This point might be used to argue that involving design researchers alongside the range of other research disciplines outlined in the A360 approach is a way forward for streamlining adoption of your five recommendations.

As a final point, I would acknowledge that while the tradition of design research with which I am most familiar can well serve the role of process evaluation as it plays out in the global health field, outcomes evaluations and cost-effectiveness studies remain more the domain of public health researchers and health economists. Such studies can be conducted relatively straightforwardly if they focus on the interventions that resulted from a design process. While good enough for most policy purposes, that approach doesn’t address the question of what proportion/aspect of good outcomes we might attribute specifically to the application of human-centered design. Given the complexity of the design process I don’t know the answer here, and while the letter raises the question, the authors’ five recommendations don’t seem to address it either. One could follow the approach taken in evaluating the impact of applying behavior change theories in the design of communication interventions - meta-analyses of many studies with and without HCD. Such an approach would likely surface a further round of questions, unaddressed in this letter, about the extent to which the impacts of design work can/should be attributed to the design practitioner’s degree of expertise, or whether a project was structured to enable the design practice to thrive, versus something integral to human-centered design as a discipline.

***Response: Having read some of the references that you mention above, we have observed that evaluation in the context of design research has focused primarily on process evaluation and there is little or no discussion of how to evaluate the impact of the interventions. This would seem to support our recommendation no.5 that impact and cost-effectiveness studies would be more efficiently designed when the intervention is defined. We agree that traditional outcome evaluation studies will not necessarily ‘address the question of what proportion/aspect of good outcomes we might attribute specifically to the application of human-centered design’. We have not addressed this question specifically in our recommendations as our primary objective was to evaluate the impact of Adolescents 360 and we did not set out to evaluate HCD per se. You raise some interesting questions around the importance of understanding the relative roles of human-centered design as a discipline, the design practitioner, and the context within the design took place. As research expands in this field we hope that these questions will be explored.***

**Minor Comments:**

It might be helpful if you clarified your use of the terms “implementer” and “designer” throughout the paper. Are you referring to implementers of the design work (i.e., designers), or to other implementers who were operationalizing the interventions that the designers designed, or both?

***Response: We have updated the text to make this clearer as follows:***

******

***‘The A360 initiative is led by Population Services International (PSI) in collaboration with the Society for Family Health (SFH) in Nigeria, design partner IDEO.org, and the Centre for the Developing Adolescent at the University of California, collectively referred to here as the ‘implementers’. The implementers were all involved in the design process. PSI and SFH are responsible for the larger scale operationalising of the designed interventions.’***

The reference below to ‘design energy’ is intriguing; I think readers would appreciate slightly more explanation of what this refers to and in what sense the term is dealt with in the cited reference.

“During intervention development the process evaluation team faced challenges as the fast-paced, highly iterative HCD process meant that the decision making process, the ‘design energy’, often went undocumented ^4^.”

***Response: ‘Design energy’ is a term that was used by IDEO and refers to how decisions were made at key points in the design process. We had included the definition in the recommendations section but we have now added earlier in the letter where we first mention the term.***

***In the cited reference (Tolley EB: Traditional Socio-behavioral research and human-centered design: Similarities, Unique Contributions and Synergies.2017), the author does not use the term ‘design energy’ but states the following which we feel represents the same idea:***

******

***‘The process of arriving at insights from fieldwork is more of a creative than scientific process, with less emphasis on creating an audit trail that can be replicated by others.’ (page 14)***

***And***

***‘Both observations and interpretations or insights from data collection activities may be simultaneously catalogued on individual sticky notes and then grouped and further re-grouped on table or wall space to look for key patterns that inform the research objectives. The process of determining what gets onto a sticky note – and what is overlooked, whether the source of the observation or insight is attributed to a particular participant or not and whether some end-user or influencer voices are (over) represented while others have no representation – is generally not described.’ (page 14)***

In the statement below, I wasn’t able to follow how starting with a blank slate may have blocked the the design team from drawing on public health evidence. Design researchers and ethnographers typically strive to approach observational fieldwork in a manner that minimizes the influence of preconceived notions, but this does not prevent them from drawing on relevant theories or evidence during the process of analysis. Was disregarding public health evidence explicitly described as typical of the design process (a point I would disagree with in general, but that may well have been true to your experience), or might it have just been another researcher to practitioner disconnect?

“Designers add value to public health as they take a different approach when addressing an issue, however, in the case of A360, a preference to work from a blank slate free of the constraints of public health evidence may have impacted on an ability to respond to the PE findings.”

***Response: We agree that the decision to work from a blank slate is not necessarily reflective of design processes and may have been specific to how IDEO chose to approach the development of A360 solutions. The HCD process, as used during the development of Adolescent 360, did not explicitly involve any review of the public health evidence for or against interventions that have been previously explored to improve adolescent uptake of modern contraceptives. Public health was one of the ‘lenses’ in the Adolescents 360 approach but our evaluation has shown that the HCD lens overshadowed the other lenses and that the public health evidence base was at times disregarded by those leading the HCD lens (see Figure 7 https://itad.com/wp-content/uploads/2019/06/A360-MTR-Report_FINAL_27th-June-2019.pdf) On reflection we have removed the highlighted sentence.***

**Typos**

“However, the iterative and flexible nature of HCD potentially poses some unique chal- lenges for evaluation ^1,3,7^.[ **remove period**] and examples of evaluations of HCD public health interventions have so far been limited ^7,8^.”

“In the ‘inspiration phase’ the designers immerse themselves with girls and **her their** influencers to understand the issues.”

“The OE study design was finalised before the interventions themselves so there remained a risk that the evaluation was not optimal.” I think this refers to Outcome Evaluation study design; it would be helpful if you could clarify on first use of the acronym.

“distinct imple- mentation phases as it was rolled **out** across Nigeria”

“because A360 was creating a new trans-disciplin **ary** approach”

“more frequent cost data draws were required during the scale-up period to capture how those change **s** might affect the “production process” and associated costs.”

***Response: Thanks. We have corrected all these typos.***
